# TRPA1 as the Intersection of Sensory Perception, Innate Immunity, and Adaptive Immune Regulation in Cutaneous Immunity

**DOI:** 10.3390/cells15191827

**Published:** 2026-10-08

**Authors:** Alessandra Torsello, Caterina Cattani, Christian Napoli, Andrea Cavani, Fernanda Scopelliti

**Affiliations:** 1 PhD School in Translational Medicine and Oncology, Department of Medical and Surgical Sciences and Translational Medicine, Faculty of Medicine and Psycology, Sapienza University of Rome, 00189 Rome, Italy; alessandra.torsello@inmp.it; 2 National Institute for Health, Migration and Poverty INMP/NIHMP, 00153 Rome, Italy; caterina.cattani@inmp.it (C.C.); christian.napoli@inmp.it (C.N.); andrea.cavani@inmp.it (A.C.)

**Keywords:** TRPA1, skin, immunity

## Abstract

Transient receptor potential ankyrin 1 (TRPA1) is a polymodal ion channel best known for its role in nociception and environmental sensing. However, TRPA1 is now recognized to be expressed in several non-neuronal cell types, including keratinocytes and immune cells, suggesting a broader role in the regulation of cutaneous immune responses. This comprehensive review examines the current evidence linking TRPA1 to cutaneous immunity under both physiological and pathological conditions with particular focus on inflammatory skin disorders such as atopic dermatitis, allergic contact dermatitis and psoriasis. The available literature reveals a complex picture in which TRPA1 can either amplify or restrain immune responses depending on the cellular context, disease setting, and nature of the triggering stimulus. Moreover, TRPA1 is dynamically regulated by cytokines, oxidative stress, and tissue-derived signals. Discrepancies among experimental studies further highlight the impact of species-specific differences, disease models, and methodological approaches on the interpretation of TRPA1 function. Through a critical appraisal of the existing literature, this review seeks to disentangle the complex and sometimes contradictory evidence surrounding TRPA1 in cutaneous immunity, identifying emerging concepts, highlighting unresolved issues, and providing a clearer framework for understanding TRPA1 biology and guiding future research.

## 1. Introduction

The skin constitutes the largest organ of the human body and serves as the first line of defense against environmental insults, including pathogens, physical injury, and chemical agents, collectively called danger signals. Beyond its role as a physical barrier, the skin hosts a highly specialized immune network composed of resident and recruited immune cells, keratinocytes, sensory neurons, and a wide range of soluble mediators that coordinate innate and adaptive immune responses [1]. The initiation and regulation of cutaneous immunity rely on a complex interplay of molecular sensors capable of detecting both microbial challenges and tissue damage [2].

Among the molecular actors involved in these processes, the Transient Receptor Potential Ankyrin 1 (TRPA1) channel has emerged as an important mediator linking environmental sensing, neurogenic inflammation, and immune regulation [3,4]. Originally characterized as a sensory ion channel expressed in nociceptive neurons, TRPA1 is now known to be present in several non-neuronal cell types, including keratinocytes [5] and immune cells [6,7,8].

The channel contains multiple regulatory and ligand-sensitive regions that enable activation through distinct mechanisms [9], including direct covalent modification by electrophilic compounds, non-covalent ligand binding, and indirect modulation by intracellular signaling pathways [10]. TRPA1 structural organization, ligand-binding sites, gating mechanisms, and pharmacological modulation have been comprehensively reviewed elsewhere [11]. Collectively, these features make TRPA1 a highly polymodal sensor capable of integrating chemical, mechanical, oxidative, and inflammatory signals [12], thereby explaining the wide range of endogenous and exogenous compounds reported to regulate its activity [13].

Nevertheless, current evidence indicates that TRPA1 expression in many skin-resident cell populations is generally low and often context-dependent, being modulated by inflammatory and pathological conditions. Moreover, differences in experimental models, disease settings, and detection techniques contribute to considerable variability across studies, thereby limiting robust comparisons of TRPA1 expression profiles among skin and immune cell types.

Given the growing evidence supporting the involvement of TRPA1 in cutaneous immune processes [4], this review focuses specifically on the skin immune responses and signaling mechanisms in which TRPA1 plays a direct or indirect role.

While several high-quality reviews have comprehensively discussed the role of TRPA1 in cutaneous disorders, the interpretation of its contribution to skin pathology remains challenging because evidence derived from immune, epithelial, and neuronal compartments is frequently discussed together. As a consequence, direct immunomodulatory functions of TRPA1 are often difficult to disentangle from neurogenic mechanisms driven by TRPA1-expressing sensory neurons. This challenge is further compounded by the fact that TRPA1 expression in many skin-resident and immune cell populations is generally low, highly context-dependent, and often assessed using heterogeneous experimental approaches, resulting in findings that are not always directly comparable across studies. Here, we provide a focused overview of the available evidence on TRPA1-mediated immunomodulation in skin physiology and disease. We critically evaluate the origin and strength of the available evidence by considering the experimental model employed, the methodology used to assess TRPA1, and the extent to which TRPA1 involvement was directly demonstrated. In doing so, we aim to distinguish observations supported by direct evidence within the cutaneous compartment from those inferred from broader experimental settings, thereby providing a clearer framework for understanding the contribution of TRPA1 to skin immune regulation.

## 2. Structure, Evolutionary Plasticity of TRPA1 and Its Translational Implications

**Figure 1:**
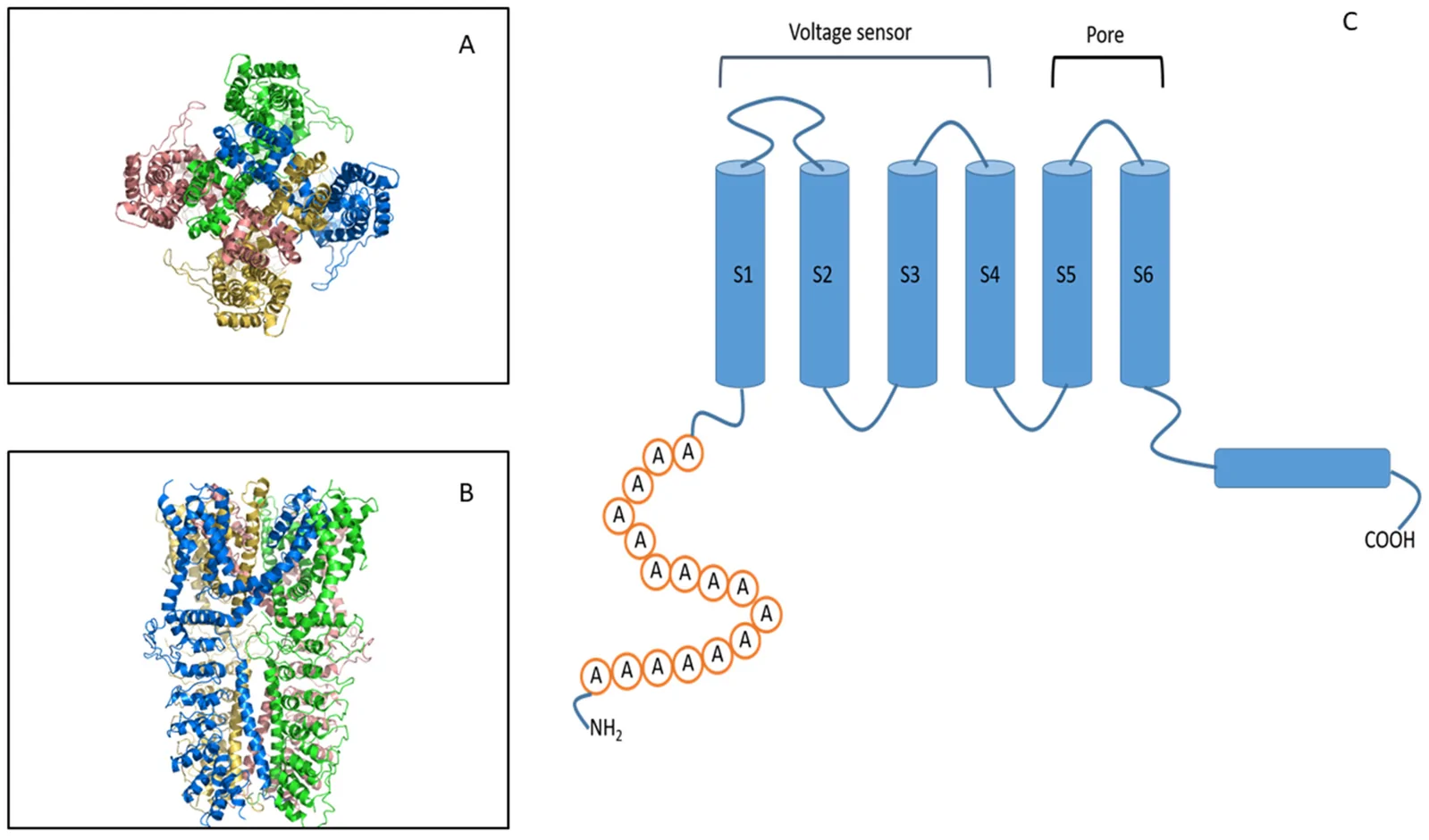
Structural features of human TRPA1. (**A**,**B**) Overall architecture of the human TRPA1 homotetramer displayed from lateral and top perspectives (PDB ID: 3J9P). (**C**) Simplified topology of a TRPA1 subunit showing the N-terminal ankyrin repeat domain (ARD), six transmembrane helices (S1–S6), the pore-forming loop between S5 and S6, and the intracellular C-terminal domain. Structural images were generated using PyMOL 3.1.0 Open-Source (1bebc9f500).

TRPA1 is a polymodal, non-selective cation channel and its activation results in Ca^2+^ influx. Beyond sensory neurons, TRPA1 is expressed by numerous structural and immune cell populations within the human skin compartment (Figure 1).

The transient receptor potential (TRP) channel family represents an evolutionarily ancient sensory system that enables organisms to monitor and respond to environmental changes [14,15,16]. Among its members, TRPA channels are considered particularly ancient, having emerged before the evolution of the vertebrate nervous system. Indeed, TRPA homologs have been identified even in early metazoans such as sponges, suggesting that their current localization in nociceptive sensory neurons is the result of later evolutionary specialization [16,17,18].

TRPA1 is thought to have originally evolved as a detector of noxious environmental stimuli, including reactive chemical compounds and temperature changes. Following the emergence and diversification of other thermosensitive TRP channels [16,19], TRPA1 may have undergone functional specialization toward the detection of chemical, mechanical, and redox-related stimuli, roles that are particularly relevant to inflammation and immune signaling [20]. As fundamental sensors of the external environment, TRP channels have been shaped by the distinct physiological and ecological requirements of different organisms [21,22]. Consequently, TRPA1 function exhibits considerable species-specific variability [23,24]. For example, evidence from murine models suggests a contribution of TRPA1 to thermosensory processes [25,26], whereas its role in human temperature perception appears limited and highly dependent on experimental conditions [27,28]. In contrast, the activation of human TRPA1 by a wide range of electrophilic and non-electrophilic compounds, both endogenous and exogenous, has been extensively demonstrated [29,30,31,32], with multiple ligand-binding and activation mechanisms having been described [11].

The interspecies variability observed in TRPA1 function is not restricted to thermosensitivity. Species-dependent differences have also been reported in the range of chemical stimuli capable of activating the channel [33,34]. Moreover, even when responsiveness to a given stimulus is conserved, the resulting functional outcome may differ across species. The same compound acts as a TRPA1 agonist in one species but as an antagonist in another [33,35,36], underscoring the extensive evolutionary diversification of both the thermal and chemosensory properties of the channel. Such differences raise questions regarding the translational value of animal models for understanding TRPA1 biology in humans. As just highlighted, substantial functional and pharmacological differences have been documented even between human and murine TRPA1 [34,37,38,39]. This issue is particularly relevant in the field of skin immunity, where the majority of in vivo studies investigating TRPA1 have been performed in mouse models of human diseases. Together, these considerations further complicate efforts to define the physiological and pathophysiological roles of TRPA1 in human health and disease.

## 3. TRPA1 in Skin Inflammation and Disease

**Figure 2:**
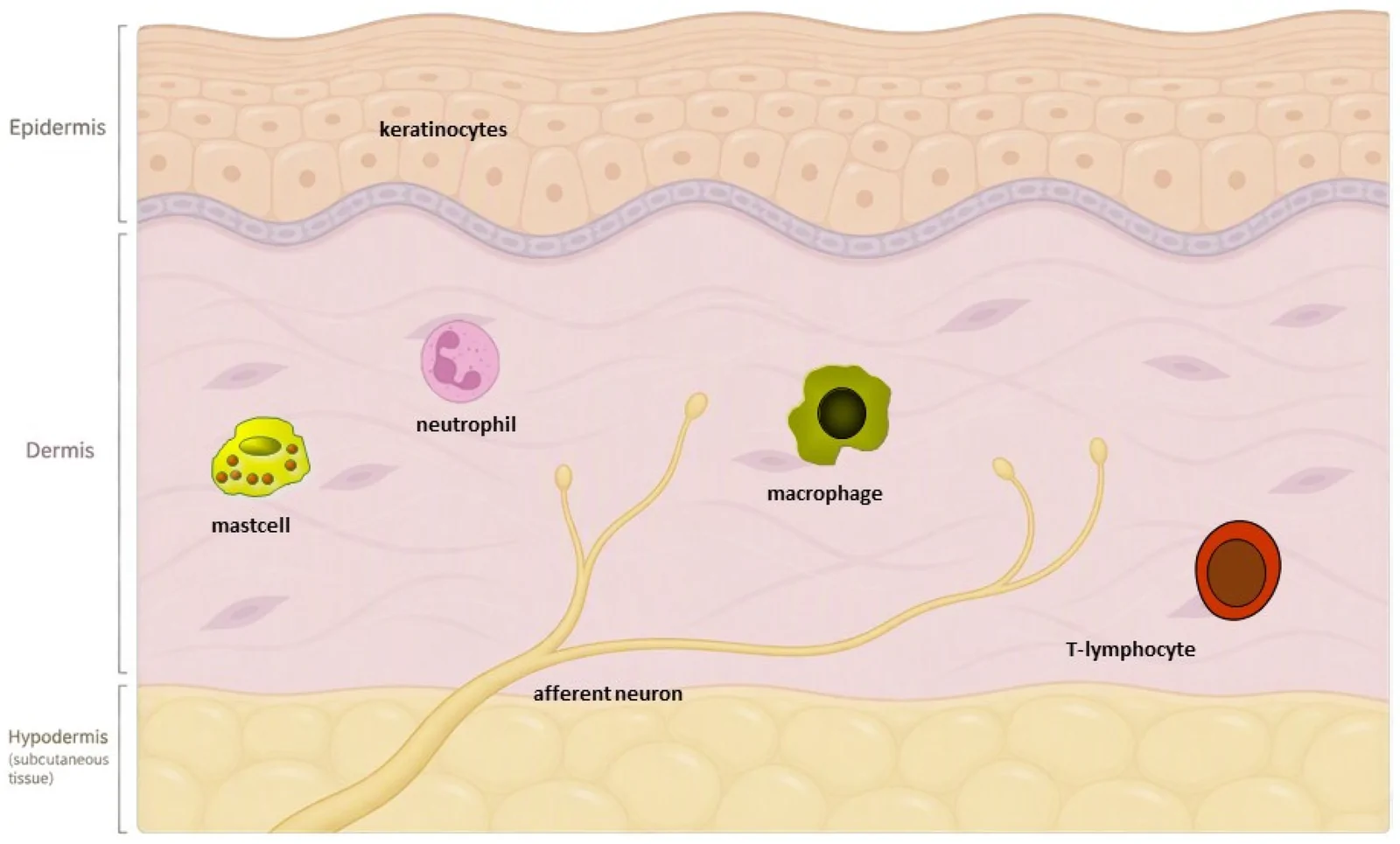
Overview of the skin cellular microenvironment. Cell populations reported to express TRPA1 and examined in studies of cutaneous immunity are shown in their typical anatomical distribution within the epidermis and dermis.

As an evolutionarily conserved sensor specialized in the detection of potentially damaging environmental cues, TRPA1 finds one of its most relevant sites of expression and function in the skin. Positioned at the interface between the host and the external environment, TRPA1 contributes not only to sensory perception but also to the orchestration of immune and inflammatory responses triggered by tissue injury, pathogens, and noxious agents. Many studies have reported increased TRPA1 expression in inflamed skin and in skin cells exposed to pro-inflammatory stimuli, both under physiological conditions associated with tissue injury and repair and in chronic inflammatory skin diseases (Figure 2) [3,4,40,41]. These observations suggest that TRPA1 is not only a regulator of cutaneous responses but is itself dynamically modulated by the inflammatory microenvironment. TRPA1 has been investigated across a wide spectrum of skin conditions, including inflammatory and autoimmune dermatoses, tissue injury and wound healing processes, and even neoplastic diseases. The role of TRPA1 in cutaneous immunity has been investigated most extensively in atopic dermatitis (AD) [42,43,44,45,46,47,48] and contact hypersensitivity (CH) models [5,49,50,51,52,53,54], but also across a broad range of chronic inflammatory and autoimmune skin disorders [4], including psoriasis [55,56,57], systemic sclerosis [58], and lichen simplex chronicus (LCS) [59], as well as in physiological and pathological responses to tissue injury, such as wound healing [39,60], blistering skin injury [61], passive cutaneous anaphylaxis [62] and sterile inflammation [63]. Despite their etiological and pathogenic heterogeneity, many of these conditions are characterized by overlapping features of inflammation, tissue injury, and altered sensory signaling, which may manifest as itch, pain, burning sensations, or other forms of cutaneous discomfort.

Among these manifestations, itch has received particular attention. As a well-established mediator of pruriceptive signaling, TRPA1 is now recognized as a key contributor to chronic pruritus, integrating signals arising from immune cells, inflammatory mediators, and damaged tissues [64]. Accordingly, a substantial body of literature has focused on elucidating the role of TRPA1 in itch initiation and maintenance and on evaluating its potential as a therapeutic target for pruritus management.

Nevertheless, the contribution of TRPA1 extends far beyond itch. Experimental studies have demonstrated that TRPA1 activity can influence multiple hallmarks of skin disease, including inflammatory mediator production, immune cell recruitment, tissue injury, epidermal remodeling, barrier dysfunction, and overall disease severity [41].

Mechanistically, TRPA1 emerges as a central signaling node linking environmental sensing and tissue damage to inflammatory and sensory responses. Its activity has been associated with neurogenic inflammation driven by sensory neuropeptide release [65,66]; activation of MAPK signaling pathways, particularly p38 and ERK [49,67]; NF-κB-dependent transcriptional programs [67]; JAK/STAT-mediated responses [46,49,68,69]; cytokine-driven immune networks involving mediators such as TNF-α [41,70], IL-1β [41], IL-6, IL-31 [48,68,71], TSLP [72] and oxidative stress-associated signaling pathways [44,73].

## 4. Cell-Specific Roles of TRPA1 in Cutaneous Immunity

In this section, we critically examine the available evidence on TRPA1-mediated immunomodulation in the cutaneous compartment. To maintain a specific focus on skin-associated mechanisms, only studies directly investigating TRPA1 modulation through pharmacological agonism/antagonism, genetic deletion, or gene silencing in skin-related experimental systems were considered. These include skin tissue samples, skin-derived cell lines, co-culture models reproducing the cutaneous microenvironment, and animal models in which TRPA1 was manipulated or assessed in the context of cutaneous stimulation or pathology. The main findings are summarized in Table 1. We also indicate whether TRPA1 expression, localization, or channel activity was directly assessed in each study, together with the methodology used. This information is particularly relevant for the interpretation of in vivo studies employing global TRPA1-deficient animals. In the absence of a direct characterization of cutaneous TRPA1 expression or activity, the observed findings cannot always be unequivocally attributed to TRPA1 signaling within skin-resident cells, as contributions from TRPA1-expressing sensory neurons may also account for the reported effects.

### 4.1. Keratinocytes

Keratinocytes are the predominant cellular component of the epidermis and play an active role in skin immunity by sensing environmental stimuli and orchestrating local inflammatory responses. The available evidence in this field is derived primarily from in vitro studies using human keratinocyte models, particularly HaCaT cells and primary human keratinocytes. Additional findings have been obtained from murine experimental systems. TRPA1 expression in keratinocytes is generally low or negligible under physiological, non-stimulated conditions [52,67]. In contrast, it becomes detectable and is often upregulated following inflammatory stimulation [43,44,70], suggesting that its expression is dynamically regulated by the local inflammatory microenvironment.

Studies in primary human keratinocytes suggest that TRPA1 contributes to the regulation of keratinocyte homeostasis by modulating both differentiation and proliferative processes. Activation of the channel induces broad transcriptional reprogramming characterized by enhanced epidermal differentiation and downregulation of proliferation-related pathways, supporting a role for TRPA1 in epidermal homeostasis [74]. Activation of TRPA1 in human primary keratinocytes has been shown to inhibit keratinocyte proliferation by inducing G2/M cell-cycle arrest [70].

TRPA1 also participates in the early sensing of and response to cutaneous allergens and irritants. Compounds such as 2,4-dinitrochlorobenzene (DNCB), 2,4-dinitrofluorobenzene (DNFB), formaldehyde, and oxazolone, commonly used to induce contact hypersensitivity in mice, are direct TRPA1 activators and promote the release of IL-1α from murine keratinocytes in an vitro model, suggesting that inflammatory response may be initiated, at least in part, through direct TRPA1 activation [5].

Similar findings have also been reported in human keratinocyte models, in which TRPA1 activation was shown to promote IL-1α and IL-1β release, supporting a conserved role for TRPA1 in the initiation of early inflammatory responses. The expression of IL-1β, IL-6, TNF-α was significantly attenuated following TRPA1 inhibition in HaCaT cells [49], indicating that activation of this channel may contribute to the maintenance of a pro-inflammatory microenvironment through the regulation of keratinocyte-derived cytokines and chemokines. Accordingly, TRPA1 activation has also been shown to promote the release of IL-1α and IL-1β by human primary keratinocytes [74].

Other evidence suggests that an inflammatory environment itself promotes TRPA1 activity in keratinocytes. In a murine model of contact hypersensitivity, co-exposure to the pro-inflammatory diisononyl phthalate (DINP) potentiated TRPA1 response through increased NFκB activity, a key regulator of TRPA1 expression in this animal model [50], as also reported in a study on HaCaT cells [67].

Furthermore, TNF-α upregulates TRPA1 in primary human keratinocytes [67,70], suggesting that TRPA1 expression or activity is enhanced by a wide range of inflammatory mediators. Collectively, these observations indicate that TRPA1 may not only respond to inflammatory stimuli but also actively contribute to the amplification of inflammatory responses.

Indeed, in HaCaT cells, TNF-α-induced TRPA1 contributes to the production of the pro-inflammatory chemokine MCP-1/CCL2, whereas its pharmacological inhibition attenuates this response, supporting a role for TRPA1 in the amplification of cutaneous inflammation [67]. Although the channel contributes to the initiation and amplification of immune responses, it has also been implicated in mechanisms that restrain inflammatory signaling. Activation of TRPA1 in primary human keratinocytes reduces the expression of molecules involved in immune activation, including HLA-DR and ICAM-1. In addition, TRPA1 stimulation decreases the production of several chemokines implicated in leukocyte recruitment, such as CXCL10, CXCL8, CCL5, and CCL20, as well as the release of Substance P [70].

Moreover, in a study employing HaCaT cells, pharmacological assays performed on hTRPA1 showed that repeated or sustained exposure to a TRPA1 agonist resulted in a progressive reduction in channel responsiveness [49]. These observations support the hypothesis that, while acute TRPA1 activation may contribute to the initiation of inflammatory responses, sustained stimulation could progressively desensitize the channel, thereby limiting its activity and attenuating inflammation.

Beyond the evidence linking TRPA1 to pro-inflammatory environment, other studies have demonstrated that TRPA1 expression is enhanced in Th2-inflammatory settings. Accordingly, attenuation of the Th2-driven inflammatory response in a reconstructed human epidermis model was associated with reduced TRPA1 expression [42]. Similarly, in a murine model of IL-13-driven atopic dermatitis, TRPA1 was markedly increased in lesional skin, with stronger immunoreactivity in keratinocytes and dermal cells, whereas only minimal expression was observed in healthy skin [43]. Taken together, the available evidence supports a model in which TRPA1 functions as an inducible sensor in keratinocytes. Although basal expression is generally low in healthy, unstimulated skin, inflammatory cues promote TRPA1 expression and activity, enabling the channel to contribute to the early initiation and subsequent amplification of cutaneous immune responses. However, the reported effects on epidermal homeostasis, immune regulation, and channel desensitization suggest that keratinocyte TRPA1 may exert context-dependent functions that extend beyond a purely pro-inflammatory role.

### 4.2. Mast Cells

Although the functional role of TRPA1 in mast cells remains less extensively characterized than in sensory neurons, accumulating evidence suggests that this channel may participate in mast cell activation, in the amplification of cutaneous inflammation, and in neuroimmune communication.

Evidence derived primarily from murine models indicate that TRPA1 expression in mast cells is dynamically regulated by the inflammatory microenvironment.

In a murine model of atopic dermatitis with transgenic mouse for IL-13, it was demonstrated that in the leucocyte infiltration, TRPA1 was predominantly expressed by mast cells, while eosinophils, Langerhans cells, macrophages, and T lymphocytes showed little to no expression. In cultured mast cells exposed to a cytokine cocktail simulating the inflammatory microenvironment of lesional skin, IL-13 emerged as the most potent inducer of TRPA1 expression, whereas IFN-γ exerted more modest effects and IL-4 downregulated channel expression [43].

In addition to cytokine-mediated regulation, TRPA1 appears to be closely linked to oxidative stress pathways. Transcriptomic analyses identified TRPA1 among the genes upregulated in human mast cell lines following exposure to reactive oxygen species (ROS). Mechanistically, ROS released by mechanically stimulated keratinocytes were shown to activate TRPA1-positive mast cells, whereas inhibition of ROS signaling reduced both TRPA1 expression and mast cell activation. Evidence from murine models corroborates these observations in human cells. Combined inhibition of ROS and TRPA1 in vivo mouse model significantly attenuated AD-like skin inflammation, reducing epidermal hyperplasia, mast cell accumulation, ROS production, and Th2 cytokine release. Together, these findings support the existence of a ROS–TRPA1 signaling axis that may contribute to the amplification of chronic skin inflammation [44].

Further evidence linking TRPA1 to mast cell responses derives from another murine model of atopic dermatitis, in which TRPA1 deficiency was associated with a reduced number of dermal mast cells in lesional skin, supporting a role for this channel in mast cell accumulation and/or maintenance within inflamed tissues [45].

Similarly, in a murine model of psoriasis, TRPA1 deficiency was associated with a marked reduction in mast cell infiltration, with mast cell numbers decreased by approximately 50% compared with wild-type mice [75].

A study demonstrated that pharmacological inhibition of TRPA1 significantly attenuated this mast cell degranulation also in a contact hypersensitivity murine model [50].

Consistent with the proposed role of mast cells in TRPA1-mediated signaling, experiments in mast cell-deficient transgenic mice revealed that the absence of mast cells was associated with reduced TRPA1 expression on dermal CGRP^+^ afferent fibers, highlighting how mast cells are important for the regulation of neuronal TRPA1 expression [43].

Overall, the available data suggest that TRPA1 expression in mast cells is regulated by both inflammatory cytokines and oxidative stress and that TRPA1-dependent mast cell activation may contribute to the persistence and amplification of cutaneous inflammation.

The relationship between TRPA1 activation and mast cell function appears to be more complex than a simple pro-inflammatory mechanism. Indeed, thymol-induced activation of TRPA1 promotes a sustained calcium influx that precedes mouse mast cell apoptosis through activation-induced cell death. Consequently, despite the initial activation of mast cells, prolonged TRPA1 stimulation ultimately reduces mast cell numbers and attenuates inflammation in a murine passive cutaneous anaphylaxis model [62]. Consistent with observations in keratinocytes, these findings indicate that sustained TRPA1 activation may engage regulatory mechanisms that limit inflammation, highlighting a role for TRPA1 that extends beyond a purely pro-inflammatory function. Overall, while the current evidence base relies predominantly on murine models, the limited data available from human mast cell studies are in agreement with the observations reported in mice. These findings suggest that the main mechanisms regulating TRPA1 expression and function in mast cells may be conserved across species, although additional studies in human systems are needed to confirm their physiological and pathological relevance.

### 4.3. Dendritic Cells

Langerhans cells and dermal dendritic cells are professional antigen-presenting cells that play a central role in cutaneous immune surveillance and in the initiation of adaptive immune responses. Direct evidence for TRPA1 expression and function in cutaneous dendritic cell subsets remains limited. In particular, while some studies have reported TRPA1 expression in dendritic cells, direct demonstration of its expression in epidermal Langerhans cells is still lacking [7]. Therefore, the specific contribution of Langerhans cells to TRPA1-mediated signaling in the skin remains to be clarified.

A canine model of atopic dermatitis provided preliminary evidence that TRPA1 is expressed by cells of the antigen-presenting compartment, as macrophage/dendritic cell populations within skin lesions displayed TRPA1 immunoreactivity [76]. Although the study did not investigate the functional significance of this expression, the findings suggest that TRPA1 may participate in inflammatory signaling pathways operating in lesional skin.

Most available evidence supports an indirect role of TRPA1 in dendritic-cell biology through neuroimmune mechanisms. Evidence from murine contact hypersensitivity models suggests that activation of TRPA1 on sensory neurons promotes dendritic cell function in the skin. TRPA1 agonists were shown to enhance skin sensitization to FITC and to stimulate the migration of FITC-bearing dendritic cells to draining lymph nodes, indicating that neuronal TRPA1 signaling facilitates dendritic cell activation and trafficking during the initiation of immune responses [51].

In a murine wound-healing model, dendritic cells may act as key intermediaries within TRPA1-dependent neuroimmune pathways. Mature dendritic cells can produce cytokines involved in type 2 inflammation, including IL-31, which induces pruritus through a mechanism requiring TRPA1 expression on sensory neurons. Similarly, in this murine model, dendritic cell-derived IL-23 has been implicated in neuroimmune pathways involving both TRPA1 and TRPV1, particularly in the regulation of γδ T-cell responses, highlighting the complexity of TRP channel signaling in cutaneous inflammation [60].

Overall, available data, which is currently limited to non-human mammalian models, suggest that dendritic cells are important downstream effectors of TRPA1-dependent neuroimmune communication rather than primary targets whose functions are directly controlled by TRPA1.

### 4.4. Macrophages

TRPA1 expression has been reported in macrophages [7]; however, relatively few studies have specifically investigated its role in macrophage-driven cutaneous immune responses. Most of the available evidence derives from animal models of skin diseases or tissue repair and frequently intersects with the study of neuroimmune interactions. Despite these limitations, current findings suggest that TRPA1 contributes to macrophage recruitment, accumulation, and polarization within the skin.

Evidence for the direct role of TRPA1 in macrophage biology comes from a murine model of bleomycin-induced scleroderma, in which TRPA1 deficiency was associated with a reduced M2 macrophage profile. Bleomycin-treated TRPA1-knockout mice exhibited lower expression of the M2 markers Arg1, MRC1, and IL-13 in the skin, while macrophages isolated from TRPA1-deficient animals showed impaired M2 polarization in vitro. Indeed, IL-4-induced expression of Arg1, MRC1, and IL-13 was significantly attenuated in TRPA1-deficient macrophages, supporting a role for TRPA1 in promoting M2 polarization and the acquisition of a pro-fibrotic phenotype [58].

A role for TRPA1 in regulating macrophage infiltration has also emerged in other skin disease models. In a murine model of atopic dermatitis, TRPA1 deficiency was associated with reduced infiltration of both M1 and M2 macrophages into lesional tissues. This effect was observed not only in TRPA1-knockout mice but also following pharmacological TRPA1 blockade, strengthening the evidence for a role of TRPA1 in macrophage recruitment and/or accumulation at sites of inflammation [45]. Consistent with these findings, reduced macrophage infiltration was also reported in a murine wound-healing model, where TRPA1-deficient mice exhibited substantially fewer macrophages in healing tissues than wild-type controls [39]. In contrast, in a murine model of psoriasis, macrophages represented the only inflammatory cell population whose abundance was not reduced in TRPA1-deficient mice. Indeed, macrophage numbers remained unchanged in the treated skin of TRPA1 KO animals, whereas the infiltration of other dermal inflammatory cell populations was significantly attenuated [75].

Beyond its role in macrophage recruitment and polarization, TRPA1 appears to participate in bidirectional neuroimmune communication. On one hand, macrophage-derived ROS have been identified as upstream activators of TRPA1 on sensory neurons in both human and murine experimental systems, suggesting that activated macrophages can directly influence neuronal function through redox-dependent signaling pathways [55]. Conversely, evidence from a murine model of psoriasis indicates that sensory neurons can regulate macrophage accumulation within skin lesions, as tissue denervation resulted in reduced macrophage infiltration. In the same study, pharmacological TRPA1 inhibition produced effects on lesion-associated discomfort comparable to those obtained through macrophage depletion, further supporting the existence of a functional interplay between TRPA1-dependent neuronal signaling and macrophage-mediated inflammation [57]. Taken together, current findings indicate that TRPA1 influences multiple aspects of macrophage biology, including tissue infiltration and M2 polarization. A recurring theme across the available studies is the close interplay between macrophages and sensory neurons. Observations from both murine and human experimental systems are broadly consistent with the existence of a conserved TRPA1-dependent neuroimmune signaling axis.

### 4.5. Neutrophils

Evidence from mouse psoriasiform inflammation models suggests that TRPA1 contributes to neutrophil recruitment within the skin. In the IMQ-induced mouse model of psoriasis, TRPA1-deficient mice exhibited markedly fewer and smaller Munro’s microabscesses compared with wild-type animals. This reduction was accompanied by decreased expression of neutrophil-associated markers and of the neutrophil chemoattractants CXCL1 and CXCL2, indicating that TRPA1 promotes neutrophilic inflammation by regulating the production of mediators involved in neutrophil recruitment and accumulation within lesional skin [75]. Consistent with these findings, pharmacological inhibition of TRPA1 in a murine model of vesicant-induced skin lesions also resulted in reduced CXCL1 expression [61]. Similarly, another study reported a reduced number of aggregates of neutrophils within the parakeratotic stratum corneum in TRPA1 knockout mice compared with wild-type controls in a murine models of contact hypersensitivity [5]. In contrast to these findings, an in vitro study on human primary keratinocytes showed that TRPA1 activation was associated with decreased CXCL8 expression [70]. Given the established role of CXCL8 in promoting neutrophil recruitment and infiltration, these observations suggest that the contribution of TRPA1 to neutrophil trafficking is not merely pro-inflammatory, but reflects a more complex immunomodulatory function within the cutaneous immune microenvironment. Such findings may also be influenced by intrinsic differences in the regulation and activity of hTRPA1 and mTRPA1. The considerable heterogeneity of the experimental models currently also precludes definitive conclusions regarding whether the observed discrepancies primarily reflect species-specific biology or model-dependent effects.

### 4.6. Lymphocytes

Evidence regarding the role of TRPA1 in lymphocytes remains heterogeneous, as it derives from different lymphocyte subsets, disease models, and experimental systems. Moreover, both direct effects of TRPA1 expression within immune cells and indirect neuroimmune mechanisms have been described, making it challenging to delineate a common TRPA1-dependent pathway across all lymphocyte populations.

In a murine model of psoriasis-like skin inflammation, confocal microscopy revealed TRPA1 expression in dermal CD4^+^ T cells, with TRPA1 staining largely localized to the plasma membrane. Interestingly, while TRPA1 colocalized with CD4, no colocalization between TRPV1 and CD4 was observed [56], highlighting TRPA1 as the predominant TRP channel detected in these cells. This finding contrasts with what is commonly observed in sensory neurons [79,80] and other human cell types [81], where TRPA1 is frequently co-expressed with TRPV1 and functionally interacts with it.

Beyond its expression, available evidence also supports a functional role for TRPA1 in T-cell-associated skin inflammation. In both acute and chronic sterile inflammation murine models, pharmacological inhibition of TRPA1 markedly attenuated the inflammatory response induced by cutaneous administration of IL-16, a cytokine released by lymphocytes and leukocytes. These findings suggest that TRPA1 contributes to IL-16-dependent inflammatory signaling and may participate in the regulation and amplification of cutaneous immune responses [63].

In a murine model of oxazolone-induced contact hypersensitivity, TRPA1 emerged as a critical regulator of both cutaneous inflammation and immune cell infiltration. Ablation or pharmacological inhibition of TRPA1 decreased the accumulation of CD4^+^ Th1 and CD8^+^ cytotoxic T cells in lesional skin, suggesting that TRPA1-dependent signaling contributes to the amplification of T cell-mediated inflammatory responses. Importantly, TRPA1 expression was primarily detected in dorsal root ganglion neurons, with transcription occurring within neuronal cell bodies rather than in infiltrating immune cells [52]. This observation supports the existence of a neuroimmune axis in which activation of neuronal TRPA1 contributes to the orchestration of local T-cell-mediated inflammatory responses.

Consistent with the finding that TRPA1 regulates immune-cell recruitment, Trpa1^−/−^ mice in a murine melanoma model displayed lower frequencies of tumor-infiltrating CD8^+^ cytotoxic T cells [77].

Conversely, TRPA1 activation in human keratinocytes decreases the expression of reduces the expression of molecules involved in T-cell recruitment and activation, HLA-DR, CXCL10, CCL5 e CCL20 and ICAM-1, suggesting that, beyond its established role in inflammation, TRPA1 may also contribute to the regulation of adaptive immune responses by modulating the recruitment and activation of lymphocytes within inflamed skin [70]. These observations are in line with a recurring theme emerging across multiple skin-resident cell populations, including keratinocytes and mast cells, in which TRPA1 appears to exert both pro-inflammatory and regulatory functions depending on the specific biological context.

TRPA1 is also functionally expressed by human mature NK cells, and its activation in vitro co-culture with melanoma cell lines promotes NK-cell activation and cytotoxicity, through increased degranulation and granzyme expression. Beyond its activating effects, TRPA1 may also exert a regulatory function in NK cells, similarly to what has been described in other cell types. TRPA1 activation has been associated with reduced CD8 expression, a change that is known to increase NK-cell susceptibility to apoptosis. Notably, this study is also the only one to date reporting TRPA1 expression in human NK cells, leaving the functional relevance of this channel in these cells largely unexplored [8].

TRPA1 has also been implicated in the recruitment of innate-like lymphocytes. In a mouse model of wound-healing in concomitant context of psoriasis, activation of TRPA1 channel after IMQ treatment promoted the recruitment of dermal γδ T cells rather than conventional αβ T cells to wound-edge tissues. Because γδ T cells constitute one of the principal sources of IL-17 in the skin, their accumulation suggested the involvement of type 17 immunity in TRPA1-mediated tissue repair. The regenerative effects of IMQ were abolished in mice with impaired IL-23 signaling, indicating that activation of the IL-23/IL-17 axis is required for TRPA1-dependent tissue regeneration [54].

### 4.7. TRPA1 Modulation of Inflammatory Skin Diseases

While the previous sections focused on cell-specific and mechanistic evidence, additional support for a role of TRPA1 in cutaneous inflammation comes from studies evaluating disease outcomes, tissue expression patterns, and the consequences of pharmacological or genetic TRPA1 modulation. Although these investigations often do not identify the precise cellular populations responsible for the observed effects, they provide important evidence linking TRPA1 activity to the overall development and severity of inflammatory skin disorders.

A recurring observation is that compounds exerting anti-inflammatory effects are often associated with a concomitant reduction in TRPA1 expression. For example, in a mouse model of atopic dermatitis, treatment with the saponin Momordin Ic markedly alleviated skin inflammation and decreased the production of pro-inflammatory mediators. These effects were paralleled by reduced TRPA1 expression, suggesting that modulation of this channel may contribute to the therapeutic response [46]. Similar findings have been described in mouse models of contact hypersensitivity following treatment with Chi-Hang solutions [53] and Qingpeng ointment [54], collectively supporting a link between TRPA1 downregulation and attenuation of cutaneous inflammation.

While anti-inflammatory treatments are frequently accompanied by reduced TRPA1 expression, direct pharmacological inhibition or genetic ablation of TRPA1 similarly attenuates inflammatory responses and decreases the production of pro-inflammatory cytokines, across a variety of murine disease models. In a mouse model of atopic dermatitis, treated TRPA1^−/−^ mice exhibited significantly lower levels of IL-1β, TNF-α, and IL-6 in skin lesions compared with treated WT mice [45].

TRPA1 activation contributes to the production of pro-inflammatory mediators also in vesicant-induced skin injury. Pharmacological inhibition of TRPA1 significantly reduced the expression of IL-1β and CXCL1/KC, cytokines and chemokines involved in the amplification of cutaneous inflammation and leukocyte recruitment. TRPA1 blockade also decreased MMP-9 levels, suggesting that TRPA1 promotes inflammatory and tissue-damaging responses following vesicant exposure [61].

In a mouse model of psoriasis, pro-inflammatory and Th17-related inflammatory cytokines such as IL-1β, IL-6, IL-23, IL-17A, IL-17F and IL-22 in the treated skin of TRPA1 KO mice were all reduced compared with WT mice [75]. Another study with mouse model of psoriasis showed elevated tissue levels of IL-17A and IL-22 as well as systemic IL-17A, whereas these responses were absent in TRPA1-deficient animals [60].

In a murine model of contact hypersensitivity, TRPA1-deficient mice exhibited reduced expression of cytokines associated with both Th1- and Th2-driven immune responses. Notably, however, the strongest effects were observed for the Th2 cytokines IL-4 and IL-13, whose induction was almost abolished [78]. Similarly, pharmacological blockade of TRPA1 in another study almost completely abolished IL-13 expression in the inflamed skin [50]. A reduction in IL-4 and IL-13 expression or production was also observed in atopic dermatitis murine models, both with TRPA1-deficient mice [45] or pharmacological blockade [44].

Further findings suggest that a Th2-dominated inflammatory environment is required for sustained TRPA1 expression in sensory afferents. Indeed, in a mouse model of atopic dermatitis, therapeutic strategies that attenuate Th2 inflammation, including the reduction in IgE and Th2 cytokines, have been shown to decrease TRPA1 expression in both the spinal cord and dorsal root ganglion neurons, supporting a mechanistic link between type 2 inflammation and TRPA1 upregulation [47].

Other pharmacological approaches modulating Th2-associated inflammatory pathways have also been shown to downregulate TRPA1 expression. In a mouse model of atopic dermatitis, treatment with fraxinellone significantly reduced the expression of IL-31 and IL-31RA, key mediators of type 2 inflammation and pruritus, while simultaneously decreasing TRPA1 and TRPV1 levels in both lesional skin and dorsal root ganglia. The parallel reduction in Th2-related cytokine signaling and TRPA1 expression further supports a link between type 2 immune responses and this channel [48].

Consistent with the evidence discussed so far, increased TRPA1 expression has also been reported in human psoriatic skin samples compared with healthy control [75]. However, although the available evidence tends to support a positive association between cutaneous inflammation and TRPA1 upregulation, this relationship is not consistent across all inflammatory skin disorder models, even when predominantly Th2-driven. Evidence from skin samples of patients with lichen simplex chronicus suggests that chronic skin disease may also be associated with a reduction in TRPA1 expression. In this condition, TRPA1 levels were decreased, particularly within the epidermal compartment, while remaining detectable in keratinocytes, vascular endothelial cells, and nerve fibers [59]. Notably, this study, together with the aforementioned investigation in psoriatic skin, represents one of only two studies discussed in this section conducted on patient-derived samples, thus evaluating human TRPA1 rather than its murine counterpart. Therefore, the reported downregulation may be attributable either to the distinct pathogenic features of the inflammatory disorder under investigation or to species-specific differences in TRPA1 regulation and function between humans and mice.

## 5. Context-Dependent and Bidirectional Regulation of Cutaneous Immunity by TRPA1

Although TRPA1 expression in the skin is generally accepted, its basal expression under physiological conditions remains low [4]. Interestingly, available evidence consistently identifies NF-κB-dependent signaling as a major mechanism driving TRPA1 induction [50,67]. This observation suggests that TRPA1 expression is linked to inflammatory cues and that the extent of its induction may depend on the specific signaling pathways engaged by different stimuli. In this regard, the study by Liu et al. [52] reported that TRPA1 expression in skin samples from mice chronically exposed to oxazolone was barely detectable. In other similar murine models of contact hypersensitivity, TRPA1 expression has generally been demonstrated [46,49,50,53,54]. However, these studies employed different inflammatory stimuli (MC903, SABDE, FITC), raising the possibility that not all inflammatory triggers induce TRPA1 expression to the same extent in murine skin. The same study further highlights that TRPA1-dependent inflammatory responses may vary substantially depending on the nature of the stimulus. For example, oxazolone treatment did not result in significant differences in IL-1β levels between wild-type and Trpa1-deficient mice, as assessed by both PCR and ELISA. In contrast, acute exposure to urushiol was associated with reduced IL-1β production in Trpa1 knockout animals [52].

These observations suggest that both TRPA1 induction and the downstream consequences of its activation are strongly influenced by the stimulus employed. Interestingly, a similar context-dependent pattern emerges in skin macrophages. While in the study of Shepherd et al. [55] electrophysiological analyses failed to reveal appreciable TRPA1 activity in murine macrophages treated with AITC, another study have demonstrated a functional role for macrophages TRPA1 in regulating infiltration and polarization following cutaneous bleomycin treatment [58].

Similar discrepancies have also emerged in studies exploring the contribution of TRPA1 to macrophage biology in skin environment. While TRPA1 deficiency reduced macrophage infiltration in murine models of scleroderma [58], atopic dermatitis [45] and wound healing [39], no comparable reduction was observed in a murine psoriasis model, where macrophages were the only major inflammatory cell population whose abundance remained unchanged following TRPA1 deletion [56]. Although initially contradictory, these findings likely reflect disease-specific differences in the mechanisms governing macrophage recruitment and function.

Notably, among the studies included in this review, reports of absent or very low TRPA1 expression despite inflammatory stimulation have been observed predominantly in murine models. Although direct comparisons are complicated by methodological differences, TRPA1 expression appears to be more consistently detected in human skin and skin-derived cells. This discrepancy may reflect well-recognized differences in the cellular composition and histological organization of murine and human skin [82], which could influence both basal expression patterns and stimulus-dependent regulation of the channel. Moreover, in some studies, TRPA1 expression and/or functionality was demonstrated in human samples, whereas mechanistic investigations of disease pathogenesis were performed exclusively in mouse models [44,67,75]. Although this approach provides important translational support for the relevance of TRPA1 in human skin, it also adds an additional variable to the interpretation of the available evidence.

The complexity arising from the integration of evidence generated across different biological systems is also evident when examining the role of TRPA1 in neutrophil recruitment. In contrast to evidence obtained from murine models, an in vitro study in primary human keratinocytes showed that TRPA1 activation reduced CXCL8 expression [70], a key mediator of neutrophil recruitment. Direct comparison between these studies is complicated not only by differences in experimental design, but also by the fact that they were conducted in distinct biological systems. While the murine studies evaluated neutrophil accumulation within an intact inflammatory microenvironment, the human study focused on the response of a single skin-resident cell population in isolation. Furthermore, CXCL8 lacks a direct murine orthologue [83]. Consequently, the apparent discrepancy may reflect both differences in chemokine biology and the use of experimental models that capture distinct aspects of TRPA1-dependent immune regulation. A similar complexity emerges when individual cell populations are examined. For instance, studies performed in primary human keratinocytes have generated seemingly opposite conclusions. Whereas one study reported anti-proliferative and anti-inflammatory effects following TRPA1 activation [70], another described a pro-inflammatory response characterized by induction of inflammatory mediators [74]. Although these findings appear contradictory, important methodological differences should be considered. Notably, the studies employed different TRPA1 agonists (icilin and AITC), neither of which was fully selective for the channel [84,85]. Consequently, at least part of the observed discrepancy may reflect differences in the pharmacological properties of the stimuli used, as well as the activation of TRPA1-independent pathways.

As the studies discussed above relied on a variety of TRPA1 agonists and antagonists, often yielding distinct biological outcomes, Table 2 provides an overview of the principal pharmacological modulators used across the literature reviewed.

The table summarizes the interaction type (agonism/antagonism) and molecular potency of the selected compounds cited in the literature reviewed herein, strictly limited to bioactive data formally indexed and available within the open-source ChEMBL database (Target ID: CHEMBL5013). Compounds lacking direct in vitro quantitative values on the isolated target within the platform (e.g., Oxazolone, IMQ, DNCB, DNFB) were excluded from this specific dataset to ensure methodological homogeneity. For each ligand, the arithmetic mean of the pChEMBL values was calculated, followed by mathematical back-transformation to express final potencies in micromolar (μM) concentrations.

A similar pattern emerges in the regulation of adaptive immune responses. In some murine models, TRPA1 promotes the recruitment and accumulation of T lymphocytes within inflamed skin [52,77]. By contrast, TRPA1 activation in primary human keratinocytes suppresses the expression of HLA-DR, CXCL10, CCL5, CCL20, and ICAM-1, molecules involved in lymphocyte recruitment and activation [70]. However, these observations may not be truly contradictory. The murine studies primarily assessed TRPA1 within an intact tissue environment, where neuronal, immune, and structural skin cells interact through complex neuroimmune and skin immune networks, whereas the human study examined cell-autonomous responses in isolated keratinocytes. The pro-inflammatory role observed in murine models may reflect not only TRPA1-dependent neuroimmune signaling but also its contribution to broader mechanisms of cutaneous immune regulation that are absent in simplified in vitro systems.

Beyond differences in species, cell type, and inflammatory stimuli, the specific pathological context itself appears to be a major determinant of TRPA1 function. Indeed, even within the same species, distinct disease models may reveal markedly different, and sometimes apparently opposing, biological roles for TRPA1.

This concept is exemplified by findings obtained in a vesicant-induced skin injury model, in which pharmacological TRPA1 inhibition ameliorated tissue damage, whereas genetic TRPA1 deletion resulted in the opposite phenotype [61]. Because no additional studies have investigated this specific model or the vesicant agent CEES, mechanistic interpretations remain speculative. Nevertheless, the divergent outcomes observed following pharmacological inhibition and constitutive genetic ablation suggest that TRPA1 may participate not only in inflammatory processes but also in regulatory or homeostatic pathways. Complete genetic deletion may therefore have broader biological consequences than pharmacological blockade of channel activity alone. Moreover, TRPA1 is often co-expressed with other receptors with which it establishes functional cross-talk [81,86,87,88]. Therefore, constitutive deletion of TRPA1 may perturb the balance of these receptor complexes in a manner that extends beyond simple channel inhibition.

Additional support for this hypothesis comes from observations in human LCS, where TRPA1 expression was reduced in lesional skin compared with healthy or non-lesional tissue. This contrasts with findings in other chronic inflammatory human skin disorders, including atopic dermatitis and psoriasis, where TRPA1 expression is commonly increased [43,75]. However, these apparently divergent findings may still be consistent with a role for TRPA1 in the maintenance of cutaneous immune and tissue homeostasis. Both excessive and insufficient TRPA1 activation may disrupt the regulatory networks governing skin inflammation, thereby contributing to disease development or progression.

## 6. Conclusions

The diversification of TRP channels allowed the progressive specialization of sensory functions [14,18,19,20,37,89]. Although human TRPA1 retains structural determinants compatible with temperature sensing [27], the presence of such molecular features does not necessarily indicate that thermosensation has been conserved as a functional role. Rather, current evidence identifies TRPA1 as a critical sensor of tissue damage and noxious environmental stimuli. By integrating chemical, mechanical, oxidative, and inflammatory signals at the host-environment interface, TRPA1 links sensory detection to immune and inflammatory responses. This unique positioning places the channel at the crossroads of neuroimmune communication and makes it an attractive target for understanding and potentially modulating cutaneous inflammatory disease.

As summarized in Table 1, the available evidence highlights a complex and context-dependent role of TRPA1 in cutaneous immunity. Depending on the cellular type involved, TRPA1 activation may influence neurogenic inflammation, cytokine production, leukocyte recruitment, antigen presentation, and tissue remodeling. Although the magnitude and direction of these effects vary according to the biological context, evidence from numerous murine disease models indicates that pharmacological inhibition or genetic deletion of TRPA1 frequently attenuates key pathological features of skin inflammation, including edema, pruritus, fibrosis, and lesion severity. One of the major challenges is defining the precise cellular origin of TRPA1-dependent effects observed in complex inflammatory settings. In many studies, neuronal, epithelial, and immune-cell contributions cannot be clearly distinguished, making it difficult to establish whether the observed responses arise directly from TRPA1 activation within immune cells or indirectly through neuroimmune interactions.

A second unresolved issue concerns the apparently opposing functions attributed to TRPA1 across different experimental settings. In some skin-resident cell populations, including keratinocytes and mast cells, TRPA1 has been implicated not only in pro-inflammatory responses but also in regulatory pathways involved in maintaining immune and tissue homeostasis. In contrast, evidence from several inflammatory disease models highlights a role for TRPA1 in the regulation of leukocyte recruitment and neuroimmune communication. TRPA1-dependent signaling has been linked to the recruitment of neutrophils, macrophages, and T lymphocytes, as well as to the amplification of inflammatory responses through interactions between sensory neurons and immune cells.

Such context-dependent nature of TRPA1 signaling is perhaps not surprising, given that the channel itself possesses intrinsic regulatory features. Indeed, TRPA1 itself can display a bimodal pattern of regulation, whereby the same compound can activate or inhibit the channel depending on its concentration [90]. This intrinsic capacity for self-modulation is consistent with the observation that TRPA1 can either promote or restrain immune responses depending on the experimental conditions. Beyond the intrinsic complexity of TRPA1 itself, dissecting its physiological role is further challenged by the extensive crosstalk among TRP channels. Stimuli capable of activating TRPA1 frequently also serve as ligands for other TRP family members that are co-expressed within the same cellular or functional network. Both stimulus detection and downstream signal processing frequently involve other co-expressed TRP channels through mechanisms of redundancy, cooperation, convergence, or functional divergence [14,26].

The diversity of endogenous and exogenous ligands capable of activating or inhibiting TRPA1, often through distinct molecular mechanisms, likely contributes to this functional heterogeneity and represents one of the major challenges in defining the precise role of this channel in skin inflammation. The species-dependent variability of TRPA1, together with the limited availability of mechanistic studies performed in human tissues, combined with the predominance of evidence derived from animal pathology models, further complicates the interpretation of existing findings and the delineation of its physiological and pathophysiological roles in humans.

Future studies should focus on resolving the cellular and mechanistic basis of TRPA1 signaling in human skin. A priority will be to determine how cell type, inflammatory context, and disease-specific microenvironments influence TRPA1 expression and function. Cell-type-specific genetic approaches, combined with advanced human-based experimental systems, will be particularly valuable for distinguishing neuronal, epithelial, and immune-cell-autonomous functions of TRPA1 during cutaneous inflammation. Human-based experimental systems, including ex vivo skin explants, human skin organoids, and advanced multicellular models, could complement the mechanistic observations obtained from animal studies and bridge the gap between experimental findings and human skin biology. While studies employing isolated primary cells have provided valuable information regarding TRPA1 function, more physiologically relevant systems are needed to investigate TRPA1 within the complex cellular and structural environment of human skin.

Future research would also benefit from the integration of advanced analytical approaches. To date, most studies investigating the role of TRPA1 in skin immunity have relied primary on conventional techniques, including PCR, Western blotting, immunofluorescence, pharmacological inhibition, and genetic deletion strategies, often assessing the impact of TRPA1 modulation on inflammatory outcomes. Although these approaches have been instrumental in establishing the involvement of TRPA1 in cutaneous inflammation, a more detailed understanding of its cellular localization, inducible expression patterns, and cell-specific functions will likely require higher-resolution analytical approaches. Advanced methodologies, including spatial transcriptomics, single-cell sequencing, and high-resolution imaging techniques, may help refine our understanding of TRPA1 distribution and regulation within the skin, while enabling a more precise characterization of its contribution to complex cutaneous immune networks. The evidence reviewed here supports the view that TRPA1 should not be regarded as a purely pro-inflammatory mediator. TRPA1 emerges as a context-dependent immunomodulator whose biological effects are shaped by cell type, inflammatory stimulus, disease setting, and tissue microenvironment. Understanding the factors that determine whether TRPA1 promotes or restrains inflammation will be critical for the rational development of future TRPA1-targeted therapeutic strategies. Indeed, TRPA1 remains an attractive target in inflammatory skin diseases. However, the complexity and context-dependency of TRPA1 signaling indicate that a more detailed understanding of its cellular and molecular functions will be required before effective therapeutic strategies can be rationally designed. In particular, future studies should clarify how TRPA1 activity is regulated across different cell populations, inflammatory settings, and disease contexts, as well as the relative contribution of neuronal and non-neuronal TRPA1 signaling to cutaneous immune responses. Such knowledge will be essential for evaluating the therapeutic potential of TRPA1 modulation in human skin disease.

## Figures and Tables

**Table 1:** Overview of experimental models and main findings of studies exploring TRPA1 involvement in cutaneous immunity. Symbols: ↑, increase/upregulation; ↓, decrease/downregulation.

Article	Main Experimental Systems	TRPA1 Modulation	TRPA1 Characterization (Main Findings)	Functional Effects of TRPA1 (Main Findings)
Jaber et al., 2026 [5]	Mouse CH model (oxazolone) Primary mouse keratinocytes Human healthy skin samples	TRPA1 KO TRPA1 antagonist (HC-030031)	TRPA1 in human basal keratinocytes and mouse CK14+ keratinocytes (RNAscope) DNFB, DNCB, formaldehyde and oxazolone induced dose-dependent Ca^2+^ influx Strong agonists covalently bound TRPA1 (molecular docking) weaker agonists showed limited/non-covalent interactions (molecular docking)	TRPA1 KO/antagonism: ↓ lesion severity (mouse model, histology) ↓ cytotoxicity (mouse primary keratinocytes, ATP-based viability assay) ↓ IL-1α release (mouse primary keratinocytes, Luminex)
Scopelliti et al., 2023 [8]	human NK cells co-cultered with human melanoma cell lines	TRPA1 agonist (AITC)	TRPA1 expression on activated NK cells (human, FACS)	TRPA1 activation: ↑ granzyme release, CD107, NKp44 (human, FACS) ↓ CD16, CD8 (human, FACS)
Murata et al., 2022 [39]	mouse WH model	TRPA1 KO	Not assessed	TRPA1 KO: ↓ granulation tissue, re-epithelialization (mouse, histology) ↓ F4/80, α-SMA (mouse, IHC, RT-PCR) ↓ Collagen 1α2 (mouse, RT-PCR)
Atoyan et al., 2009 [74]	Primary human epidermal keratinocytes	TRPA1 agonist (Icilin)	TRPA1 expression in keratinocytes (human, RT-PCR, WB) Basal epidermal localization (human, IF)	TRPA1 activation: ↑ IL-1α, IL-1β, BMP7/GDF15, p21, HSP27 (human, RT-PCR) ↓ HSP90, cdc2 (human, RT-PCR)
Cattani et al., 2026 [70]	Primary human epidermal keratinocytes Human skin sample	Not assessed	↑ TRPA1 expression after IFN-γ/TNF-α treatment (human, FACS) TRPA1 expression in basal keratinocytes (human, IHC)	TRPA1 activation: ↓ Keratinocyte proliferation (human, CyQuant) ↓ HLA-DR (human, FACS) ↓ ICAM1, CXCL10, CCL5, CCL20, CXCL8, substance P (human, ELISA)
Ju et al., 2023 [49]	Mouse CH model (SADBE) Mouse acute itch model (CQ) Human keratinocytes (HaCaT)	TRPA1 antagonist (HC-030031) TRPA1 silencing (siRNA) TRPA1 ligand (KB)	↑ TRPA1 in lesions and co-localized to K14+ keratinocytes (mouse, IF) Compounds in KB bind TRPA1 (molecular docking) Compounds in KB have activation-desensitization profile on TRPA1 (whole cell patch clamp)	TRPA1 antagonism: ↓ IL-1β, TNF-α, IL-6, IL-33, CCL2 (human, RT-PCR) ↓ lesion severity (clinical, H&E), inflammatory infiltration (mouse, Histol) ↓ scratching behavior (mouse) KB treatment: ↓ IL-1β, TNF-α, IL-6 (human, RT-PCR, reversed by siTRPA1) ↓ p-p38, p-STAT3 (human, WB, reversed by siTRPA1)
Kang et al., 2017 [50]	mouse CH model (FITC)	TRPA1 antagonist (HC-030031)	↑ TRPA1 after inflammatory stimuli (RT-PCR, IHC) NF-κB antagonism ↓ TRPA1 expression (RT-PCR, IHC)	TRPA1 antagonism: ↓ IL-13, IL-6 (mouse, IHC), mast cell degranulation (mouse, IHC), ↓ lesion severity (mouse, Histol)
Luostarinen et al., 2021 [67]	human Keratinocytes (HaCat) mouse	TRPA1 antagonist (HC-030031) TRPA1 KO	Minimal basal TRPA1 (HaCat, RT-PCR) TNF-α ↑ TRPA1 (HaCaT, RT-PCR, WB), attenuated by DEX and NFAT inhibitors (HaCaT, RT-PCR) ↑ TRPA1 via NF-κB and MAPK (HaCaT, RT-PCR)	TRPA1 antagonism/KO: ↓ MCP-1 (human, mouse, ELISA)
Swindell et al., 2021 [42]	AD-like premature reconstituted human epidermis tissues (HaCat)	Not assessed	↓ TRPA1 expression after anti-inflammatory treatment (human, RT-PCR)	Not assessed
Oh et al., 2013 [43]	IL-13 transgenic mouse model of AD human AD skin biopsy	TRPA1 antagonist (HC-030031)	↑ TRPA1 in epidermal, dermal region and dermal afferents of AD lesions (mouse, IHC) ↑ TRPA1 in keratinocytes, dermal region and dermal afferents of AD lesions (human, IHC) TRPA1-positive mast cells in close proximity of afferent nerve fibers (human, mouse IHC) ↓ TRPA1 in dermal afferents and DRG neurons in mast cell deficiency (mouse, IHC) IL-13-induced ↑ TRPA1 in skin nerve fibers (mouse, IHC)	TRPA1 antagonism: ↓ pruritus (mouse)
Hu et al., 2025 [44]	human mast cell lines treated with ROS produced by human keratinocytes (HaCaT) mouse AD model (MC-903)	TRPA1 antagonist (HC-030031)	↑ TRPA1 expression (human mast cell, RNA-seq)	TRPA1 antagonism: ↓ lesion severity, scratching (mouse) ↓ TPS, IL-4, IL-13, TNF-α (mouse, ELISA)
Zeng et al., 2021 [45]	mouse AD mode (DNCB)	TRPA1 antagonist (HC-030031) TRPA1 KO	Not assessed	TRPA1 KO: ↓ lesion severity (mouse, dermatitis score, Histol) ↓ mast cells (mouse, toluidine blue staining) and macrophage infiltration (mouse. IF, WB) ↓ IL-1β, TNF-α, IL-6, IL-4, IL-13 (mouse, RT-PCR) TRPA1 antagonism: ↓ lesion severity (mouse, clinical, Histol) ↓ scratching (mouse)
Zhou et al., 2019 [75]	human skin sample mouse PsD model (IMQ)	TRPA1 KO	↑ TRPA1 in psoriatic lesions (neutrophils, keratinocytes in spinous and basal layer (human, RT-PCR, IHC)	TRPA1 KO: ↓ lesion severity (mouse, Histol) ↓ S100A8, S100A9, CXCL1, CXCL2 (mouse, RT-PCR) ↓ T cells and mast cells infiltration (mouse, IHC) ↓ IL-17A/F, IL-22, IL-23, IL-1β, IL-6 (mouse, RT-PCR)
Wechsler et al., 2014 [62]	mouse PCA model mouse cultured mast cells	TRPA1 ligand (Thymol)	Thymol is an activator of TRPA1	TRPA1 activation: ↑ mast cell apoptosis (mouse, FACS)
Ciocchetti et al., 2022 [76]	canine AD model	Not assessed	TRPA1-postive dendritic cells (dog, IF) TRPA1-positive macrophages (dog, IF) TRPA1-positive mast cells (dog, IF)	Not assessed
Shiba et al., 2012 [51]	mouse CH model (FITC)	TRPA1 agonist (DPB, CNA, menthol, carvacrol, AITC)	Not assessed	TRPA1 activation: ↑ DC trafficking (mouse, FACS)
Wei et al., 2020 [60]	mouse WH model	TRPA1 ligand (IMQ) TRPA1 KO	IMQ effects are only partially TRPA1-dependent	TRPA1 KO: ↓ scar formation (mouse, Histol) ↓ IL-23, IL-17A, IL-22 (mouse, ELISA)
Mäki-Opas et al., 2023 [58]	mouse scleroderma model (bleomycin)	TRPA1 KO	Not assessed	TRPA1 KO: ↓ Dermal thickening (mouse, Histol) ↓ TGF-β, CTGF, fibronectin-1, YKL-40 (mouse, RT-PCR) ↓ COL1A1, COL1A2, COL3A1, Arg1, MRC1, IL-13 (mouse, RT-PCR)
Shepherd et al., 2018 [55]	mouse psoriasis model (Aldara) human DRG neurons mouse DRG neurons	A967079 (antagonist)	TRPA1 in normal and denervated skin samples (mouse, RT-PCR) ↑ ROS/RNS by skin macrophages mediate TRPA1 activation on DRG neurons (mouse, human, electrophysiology)	TRPA1 antagonism: ↓ ROS/RNS-induced activation of DRG neurons (mouse, human, electrophysiology)
Kodji et al., 2019 [57]	psoriasis mouse model (Aldara)	A967079 HC-030031 (antagonist)	TRPA1 in normal and denervated skin samples (mouse, RT-PCR)	TRPA1 antagonism: ↓ discomfort behaviors
Achanta et al., 2018 [61]	mouse ear vesicant model (CEES)	A967079 HC-030031 (antagonist)	Not assessed	TRPA1 antagonism: ↓ lesion severity (mouse, Histol), ↓ IL-1β, CXCL1/KC, MMP-9 (mouse, ELISA) TRPA1 KO similar or ↑ lesion severity (mouse, histol)
Kemény et al., 2018 [56]	mouse psoriasis model (IMQ) human keratinocytes (HaCaT)	A967079 (antagonist)	IMQ activate TRPA1, contrasted by antagonist (human, scintillation counter, FACS) TRPA1 expression on CD4+ dermal and no TRPV1 (mouse, IHC)	TRPA1 KO or antagonist: ↑ lesion severity (mouse, clinical, histol), scratching (mouse) TRPA1 KO: ↑ IL-1β, TNF-α, IL-22 (mouse, RT-PCR)
González-Rodríguez et al., 2024 [63]	mouse model	TRPA1 antagonist (HC-030031)	Not assessed	TRPA1 antagonism: ↓ IL-16-induced hyperalgesia (mouse, hot plate test)
Liu et al., 2013 [52]	mouse CH model (oxazolone, urushiol) HEK293 cells transfectec with hTRPA1 human skin sample	HC-030031, A967079 (antagonist) TRPA1 KO	Oxazolone activate TRPA1 (human, Ca2+ microscopy) TRPA1 very low expressed in skin samples (human, mouse, RT-PCR)	TRPA1 KO: ↓ lesion severity (mouse, clinical, histology), scratiching (mouse) ↓ IL-6, CXCL-2, IL-4, NGF, 5-HT (mouse, ELISA) ↓ CXCL-2, SP, NGF (mouse, RT-PCR) ↓ IL-1β after urushiol challenge (mouse, ELISA) ↓ filaggrin, loricrin, involucrin (mouse, Histol, IF) TRPA1 antagonism: ↓ recruitment CD4+ and CD8+ cells (mouse, FACS) ↓ scratching (mouse)
Forni et al., 2021 [77]	mouse melanoma model	TRPA1 KO	Not assessed	TRPA1 KO: ↓ tumor growth, ↑ pool circulating lymphocytes (mouse, hemocytometer) ↓ CD8+ cells in tumor microenvironment (mouse, FACS) ↓ oxidative metabolism in CD8+ cells (mouse, seahorse) ↑ citotoxic capacity of CD8+ cells (mouse, RT-PCR) ↑ IL-1β, IL-6, IFN-g in tumor stroma (mouse, RT-PCR)
Yang et al., 2026 [46]	mouse AD model (MC903)	not assessed	↓ TRPA1 after anti-inflammatory treatment (mouse, IF, WB)	not assessed
Yang et al., 2022 [53]	mouse CH model (SADBE)	not assessed	↓ TRPA1 after anti-inflammatory treatment (mouse, RT-PCR)	not assessed
Gong et al., 2026 [54]	mouse CH model (SADBE)	not assessed	↓ TRPA1 after anti-inflammatory treatment (mouse, RT-PCR)	not assessed
Mäki-Opas et al., 2023 [78]	mouse CH model (DNCB) human keratinocytes (HaCat)	TRPA1 antagonist (HC-030031) TRPA1 KO	Not assessed	TRPA1 KO: ↓ IL-4, IL-13, IL-19, IFN-γ (mouse, RT-PCR) TRPA1 antagonism: ↓ SRXHN1, HMOX1 (human, RT-PCR)
Zhao et al., 2023 [47]	muse AD model (DNCB)	not assessed	↓ TRPA1 after anti-inflammatory treatment (mouse, RT-PCR, WB)	not assessed
Yang et al., 2024 [48]	mouse AD model (DNFB) human keratinocytes (HaCat)	not assessed	↓ TRPA1 after anti-inflammatory treatment (human, mouse, RT-PCR, WB)	not assessed
Qiu et al., 2020 [59]	human skin sample	not assessed	↓ TRPA1 in LSC lesions (human, IHC, WB)	not assessed

**Table 2:** Pharmacological profile and quantitative potency values (EC_5__0_/IC_5__0_) of ligands targeting the human TRPA1 receptor cited in the literature reviewed.

Compound Name	Pharmacological Profile	Binding Mode	Parameter	pChEMBL (Mean)	Potency (μM)
A967079	Antagonist	Non-covalent	IC_50_	7.08	0.0838
HC-030031	Antagonist	Non-covalent	IC_50_	5.25	5.6
AITC	Agonist	Covalent	EC_50_	5.44	3.65
Cinnamaldehyde	Agonist	Covalent	EC_50_	4.87	13.5
Menthol	Agonist	Non-covalent	EC_50_	4.52	30.2
Icilin	Agonist	Non-covalent	EC_50_	4	100
Thymol	Agonist	Non-covalent	EC_50_	4.55	28.2
Carvacrol	Agonist	Non-Covalent	EC_50_	4.9	12.6

## Data Availability

No new data were created or analyzed in this study. Data sharing is not applicable to this article.

## Abbreviations

The following abbreviations are used in this manuscript:

TRPA1	Transient receptor potential ankyrin 1
LSC	Lichen simplex chronicus
ROS	Reactive oxygen species
CH	Contact hypersensitivity
AD	Atopic dermatitis
PCA	Passive cutaneous anaphylaxis
DNCB	Dinitrochlorobenzene
DNFB	Dinitrofluorobenzene
CEES	2-chloroethyl ethyl sulfide
KB	Ke-teng-zi
TRPV1	Transient receptor potential vannilloid 1
TRP	Transient receptor potential
IF	Immunofluorescence
IHC	Immunohistochemistry
FACS	Fluorescence-activated cell sorting
WB	Western blot
SADBE	Squaric acid dibutyl ester
IMQ	Imiquimod
DEX	Dexamethasone
NFAT	Nuclear factor of activated T cells
CQ	Chloroquine
WH	Wound healing
AITC	Allyl isothiocyanate
CNA	Cynnamaldehide
FITC	Fluorescein isothiocyanate
DINP	Diisononyl phthalate
